# Plant Carbohydrate Binding Module Enhances Activity of Hybrid Microbial Cellulase Enzyme

**DOI:** 10.3389/fpls.2012.00254

**Published:** 2012-11-19

**Authors:** Caitlin S. Byrt, Ricky Cahyanegara, Christopher P. L. Grof

**Affiliations:** ^1^Australian Research Council Centre of Excellence in Plant Cell Walls, Waite Campus, University of AdelaideAdelaide, SA, Australia; ^2^School of Environmental and Life Sciences, University of NewcastleNewcastle, NSW, Australia

**Keywords:** cellulase binding module, cellulose, endoglucanase, exo-glucanase, glycosyl hydrolase

## Abstract

A synthetic, highly active cellulase enzyme suitable for *in planta* production may be a valuable tool for biotechnological approaches to develop transgenic biofuel crops with improved digestibility. Here, we demonstrate that the addition of a plant derived carbohydrate binding module (CBM) to a synthetic glycosyl hydrolase improved the activity of the hydrolase in releasing sugar from plant biomass. A CEL-HYB1-CBM enzyme was generated by fusing a hybrid microbial cellulase, CEL-HYB1, with the CBM of the tomato (*Solanum lycopersicum*) SlCel9C1 cellulase. CEL-HYB1 and CEL-HYB1-CBM enzymes were produced *in vitro* using *Pichia pastoris* and the activity of these enzymes was tested using carboxymethylcellulose, MUC, and native crystalline cellulose assays. The presence of the CBM substantially improved the endoglucanase activity of CEL-HYB1, especially against the native crystalline cellulose encountered in *Sorghum bicolor* plant cell walls. These results indicate that addition of an endogenous plant derived CBM to cellulase enzymes may enhance hydrolytic activity.

## Introduction

Increasing the efficiency of enzymatic hydrolysis of cellulose is a key challenge in the process of reducing the cost of producing transport fuels from lignocellulosic biomass (Cheng et al., 2011). Cellulase enzymes are required for cellulose hydrolysis because the β-1,4 linkage between glucose molecules in cellulose is a very stable bond that has a half-life of approximately five to eight million years at room temperature (Wolfenden and Snider, 2001). One factor influencing the efficiency of hydrolysis is the interaction of soluble cellulase enzymes with the insoluble cellulose (Cheng et al., 2011). Cellulase enzymes belong to different families of glycoside hydrolases (GHs). Many cellulases contain a catalytic domain (CD) and a cellulose binding domain or module (Linder and Teeri, 1997); these are connected by a Pro/Ser/Thr rich linker (Batista et al., 2011). Other domains may also be present and these were recently described for bacterial cellulases in a comprehensive genome analysis (Medie et al., 2012).

There are three types of cellulase enzymes; endo-β-1,4-glucanases (EC 3.2.1.4), exo-β-1,4-glucanases (EC 3.2.1.91, cellobiohydrolases), and β-glucosidases (EC 3.2.1.21; Taylor et al., 2008). Endo-β-1,4-glucanases cleave the β-1,4 bond between glucose residues in cellulose microfibrils randomly, whereas cellobiohydrolase hydrolyzes the bond at the reducing or non-reducing end of the polymer. Cellobioses are the product of the reaction catalyzed by these enzymes. The β-glucosidase cleaves cellobioses to produce monosaccharides that are fermentable by *S. cerevisiae* to produce ethanol (Louime and Uckelmann, 2008).

Carbohydrate binding modules (CBMs) target an enzyme to a specific component of the cell wall (Medie et al., 2012) and the hydrolysis of cellulose by a cellulase enzyme is initiated by the interaction of the cellulose binding module with the cellulose fiber (Caparrós et al., 2012). CBMs facilitate binding of the CD but they are not required for processivity; thus, they may increase activity of an enzyme on crystalline substrates but rarely increase activity on substrates such as carboxymethylcellulose (CMC) (Wilson, 2012).

There are three different states in which an enzyme with a CD and CBM may exist in the presence of crystalline substrate. The enzyme may be free in solution, the CBM may be bound but the CD unbound or both the CBM and the CD may be bound (Kostylev et al., 2011). The most common state is where the CBM is bound and the CD is unbound (Kostylev et al., 2011). This indicates that the binding of the enzyme via the CBM and the initial cleavage are two distinct events (Kostylev et al., 2011).

Carbohydrate binding modules may affect the thermostability of the CD activity, enabling enzymes to maintain activity at high temperatures (Mingardon et al., 2011). Three of the amino acid residues that are important for the thermostability activity of CBMs were identified in a recent study by using site-directed mutagenesis of a *Bacillus* sp. family 9 endoglucanase with a family 3 CBM (Yin et al., 2011). These residues may form hydrophobic stacking interactions with non-polar sugar rings of substrates; thus, the flexibility of the covalent link between the CBM and CD, and the polarity of this linkage is important (Yin et al., 2011).

### Genetic engineering of CBM

Cellulase enzymes may provide solutions for industrial applications such as biomass processing for biofuels (Wilson, 2012) and detergency (Caparrós et al., 2012). Furthermore, the *in planta* expression of heterologous cellulase enzymes may reduce bioethanol production costs. Here we describe the addition of a plant derived CBM to a synthetic hybrid microbial glycosyl hydrolase enzyme that is codon optimized for expression *in planta*.

## Materials and Methods

### Generation of the synthetic glycosyl hydrolase and isolation of *SlCel9C1*

The *CEL-HYB1* gene described by Xue et al. (2003) was synthesized by GENEART (GENEART gene synthesis service, Regensburg, Germany). *CEL-HYB1* was then amplified to incorporate *Eco*RI and *Pst*I restriction sites at the 5′ and 3′ end of the gene respectively. This product was ligated into the pPICZαA vector and confirmed by sequencing.

RNA was extracted from 14-day-old *S. lycopersicum* cv Moneymaker hypocotyls using an RNeasy^®^ Plant Mini Kit (Qiagen). First strand cDNA was synthesized from the isolated RNA using a QuantiTect^®^ Reverse Transcription Kit (Qiagen). The cDNA encoding the cellulase SlCel9C1 (GenBank ID AF098292; see Urbanowicz et al., 2007) was amplified using the forward and reverse primers 5′-TTCCGTCGTTACAACCCG-3′ and 5′-AGTTGCCCTTTGTTGAATAGT-3′. The *Pst*I and *Not*I restriction sites were incorporated at the 5′ and 3′ ends of the amplicon respectively. A three-way ligation reaction fused the *Pst*I digested CBM encoding gene to the 3′ end of the *Pst*I digested *CEL-HYB1*. The fusion gene was inserted into the *Eco*RI-*Not*I site of the pPICZαA vector (Invitrogen). The pPICZαA_*CEL-HYB1* and pPICZαA_*CEL-HYB1-CBM* were linearized with *Pme*I and electroporated into *Pichia pastoris* (*P. pastoris*). Transformed cells were checked by colony PCR to confirm the presence of the respective vectors and positive clones were re-cultured for subsequent analysis.

### Enzyme production in *Pichia pastoris*

The expression vector, pPICZαA (Invitrogen) directed the heterologous protein to be secreted using the α-factor signal peptide and was tagged with both c-myc epitope and 6X histidine residues. Throughout the development of the constructs, PCR with appropriate primer sets was employed to amplify the required gene without the native signal peptides. The genes were then fused in frame with the α-factor secretion signal. The signal peptide is removed by kex2 and ste13 proteases after it attaches to a receptor releasing mature protein. During the generation of the *P. pastoris* cell lines capable of producing the cellulase enzymes, PCR was performed to confirm the success of the yeast transformation procedure.

*P. pastoris* SMD1168 cells were grown on yeast peptone dextrose (YPD) solid media whereas electroporated cells were grown on YPD (YPDS) media supplemented with sorbitol and zeocin antibiotics (100 μg mL^−1^). The yeast cells were then incubated at 30°C for 3–4 days in the dark. *P. pastoris* cells used in protein overproduction were cultured in buffered glycerol-complex medium (BMGY) and buffered methanol complex medium (BMMY) at 30°C for 4 days whilst shaking at 250 rpm. Colonies transformed with the desired expression vectors (pPICZαA_*CEL-HYB1* and pPICZαA_*CEL-HYB1-CBM*) were cultured in BMGY to OD_600_ of 2–6 then collected cells were resuspended in BMMY to an OD_600_ of 1.0 and grown for a further 4 days. Induction was maintained by adding 100% methanol to a final concentration of 0.5% each day. Samples were collected prior to the induction and then 6, 12, 24, 48, 72, and 96 h after induction. The culture was centrifuged and protein secreted into the culture medium was concentrated using Centricon Plus^®^-70 columns (Millipore). Heterologous protein was detected by Western blot. Protein samples were separated electrophoretically (200 V for 30 min) on a 10% SDS polyacrylamide gel. Protein bands on a replica gel were detected by staining with Coomassie Brilliant Blue stain [0.25% (w/v); Methanol 20% (v/v); Acetic acid 10% (v/v)] and staining was then removed with Coomassie destain solution [Methanol 20% (v/v); Acetic acid 10% (v/v)]. Separated proteins were transferred (30 V at 4°C overnight) onto a nitrocellulose HybondTM – C Extra membrane. The nitrocellulose membrane was washed with 1× TBST three times and blocked with 1× TBST/5% skim milk/0.05% Tween 20 solution for 1 h. The blocked membrane was incubated for 1 h with a primary polyclonal antibody raised against a c-myc protein epitope in rabbit. The primary antibody was diluted 1:5000 in 1× TBST/5% skim milk/0.05% tween 20 solution. The membrane was washed three times with 1× TBST and incubated with anti-rabbit secondary antibody conjugated to alkaline phosphatase (Sigma), diluted 1:5000, for 1 h. The membrane was washed three times in 1× TBST solution and the band visualized with Western Blue^®^ standard substrate for alkaline phosphatase (Promega). Recombinant protein was quantified using the Bradford protein assay and diluted appropriately for further assays.

### CMC and MUC assays

The CMC assay was modified from Ghose (1987) and Miller (1959). The recombinant cellulase enzyme was diluted to the required concentrations with 0.05 M sodium citrate buffer at pH 6.0 in a volume of 500 μL and incubated at the required temperature for 5 min. Five hundred microliters of 2% CMC was added and the sample incubated for a further 30 min. Three milliliters of dinitrosalicylic acid (DNS) reagent was added and samples incubated at 100°C for 15 min. One milliliter of 40% potassium sodium tartrate was added and then samples were diluted with 19 mL of Milli-Q water. The optical density of the samples was measured immediately at 540 nm. This value was used to determine the concentration of cellulase required to release reducing sugar equivalent to 0.5 mg of glucose. A d-glucose solution prepared at various concentrations was used as the standard. A blank was prepared by adding the enzyme to the substrate just prior to the heating step. The activity of the cellulase enzyme was expressed as μmol of reducing sugar min^−1^(U) mg^−1^.

Cellobiohydrolase activity of the recombinant proteins was quantified using 4-methylumbelliferyl-β-d-cellobioside (MUC; Sigma) as a substrate in an assay modified from Ziegelhoffer et al. (2001). Appropriate dilutions of cellulase enzyme were initially made using 0.05 M of sodium acetate pH 6.0 and 0.1 M sodium chloride. One microliter of the dilutions was added into 100 μL of MUC reaction buffer in a sealed microtiter plate and incubated for 30 min at 37°C. The hydrolysis reaction was terminated by adding 100 μL of 0.15 M glycine (pH 10) and the mixture was further incubated at room temperature for 5 min. Relative fluorescence at 460 nm was measured using an excitation wavelength of 355 nm (POLARstar Omega, BMG Labtech). A blank was prepared by adding the enzyme to the substrate just prior to the addition of glycine. The activity of the cellulase enzyme was then calculated using a standard curve prepared previously using 4-Methylumbelliferone, the product of the MUC hydrolysis reaction. The activity of the enzyme was expressed as μmol of MU min^−1^ (U) mg^−1^.

### Native crystalline cellulose assay

The substrate, milled stem tissue of *Sorghum bicolor* (variety MR-Buster), was pre-treated in a manner similar to that described in Vancov and McIntosh (2012); with 1% H_2_SO_4_ for 90 min at 121°C. Pre-treated material was washed four times with Milli-Q water before use in the method described for CMC. Cellulase enzymes were incubated with the substrate for 20 h at the optimal pH and temperature for the respective enzymes. A blank was prepared by adding the enzyme to the substrate just prior to the heating step. The activity of the cellulase enzyme was expressed as μmol of reducing sugar min^−1^(U) mg^−1^.

## Results

### Engineering of a glycosyl hydrolase enzyme

**Figure 1:**
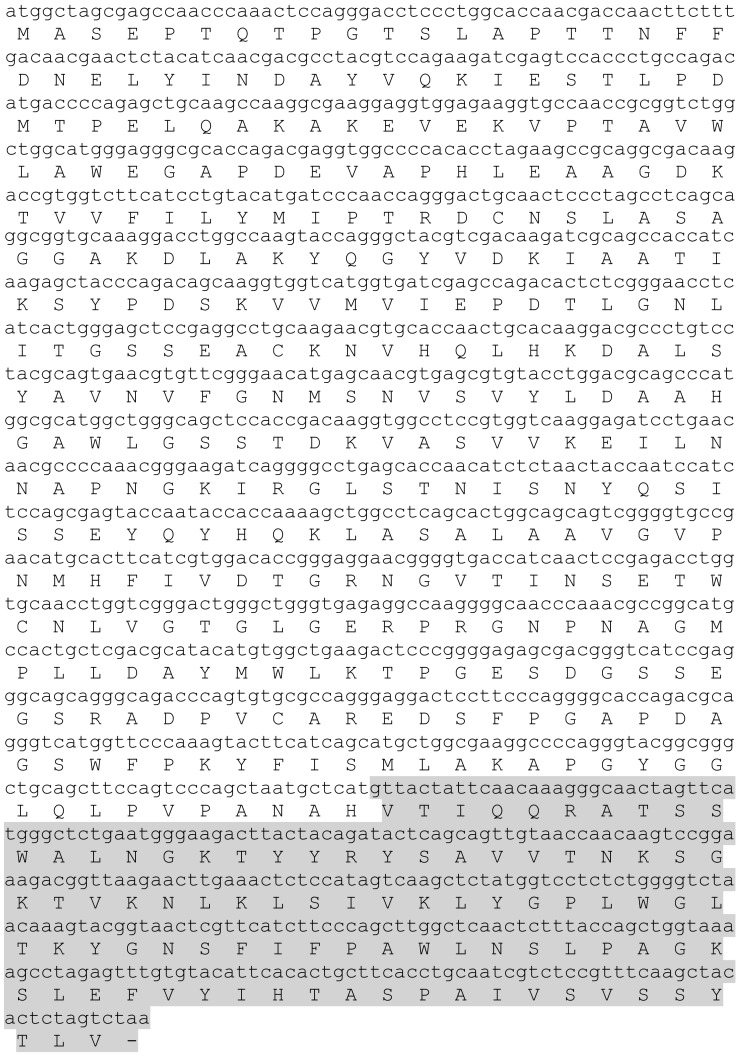
**Nucleotide and amino acid sequence of the synthetic glycosyl hydrolase**. The synthetic glycosyl hydrolase CEL-HYB1 (unshaded) is a hybrid molecule with fragments from *Neocallimastix patriciarum* and *Piromyces* sp. (Xue et al., 2003) and the carbohydrate binding module (CBM; shaded) is from the tomato CEL9 (*Solanum lycopersicum*).

In order to develop a highly active glycosyl hydrolase suitable for production *in planta* the nucleotide sequence of a highly active endo-β-1,4-glucanase of microbial origin (a hybrid from *Neocallimastix patriciarum* and *Piromyces* sp.), previously codon optimized for plants (Xue et al., 2003) was fused directly to the nucleotide sequence encoding a CBM of plant origin (from tomato SlCel9C1; Figure 1). Native enzymes often contain a Pro/Ser/Thr rich linker region between the CD and the CBMs (Batista et al., 2011); however, no linker region was included between CEL-HYB1 and the CBM in this study.

### *In vitro* production of CEL-HYB1 and CEL-HYB1-CBM

**Figure 2:**
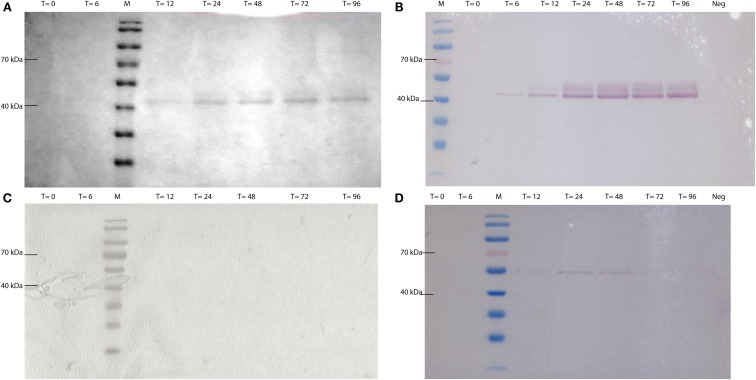
**CEL-HYB1 and CEL-HYB1-CBM visualized by SDS-PAGE and Western blotting**. Protein production from *Pichia pastoris*_CEL-HYB1 **(A)** and *P. pastoris*_CEL-HYB-CBM **(C)** induced with 0.5% methanol; heterologous protein excreted in the supernatant was visualized using SDS-PAGE and Coomassie Brilliant Blue staining. Production of CEL-HYB1 **(B)** and CEL-HYB1-CBM **(D)** was confirmed by Western blot with the c-myc antibody. Culture supernatant was harvested at the specified times (*T* = 0, 6, 12, 24, 48, 72, and 96 h; Neg = negative control; M = PageRuler™, Fermentas) after induction with 0.5% methanol.

CEL-HYB1 and CEL-HYB1-CBM were produced *in vitro* using a *Pichia pastoris* expression system. The expression vector used, pPICZαA containing the methanol inducible *AOX1* promoter, secreted heterologous protein (CEL-HYB1 or CEL-HYB-CBM) into the culture supernatant using the α-factor signal peptide. Supernatant was concentrated using Centricon Plus^®^-70 columns (Millipore) prior to SDS-PAGE and Coomassie Brilliant Blue staining (Figures 2A,C). Western blotting confirmed the presence of a band corresponding to the CEL-HYB1 at approximately 42 kDa and approximately 55 kDa for CEL-HYB1-CBM (Figures 2B,D). The concentration of CEL-HYB1 protein reached a maximal level at 72 h whereas CEL-HYB1-CBM protein production was relatively low and efflux to the supernatant peaked 24 h after methanol induction.

**Figure 3:**
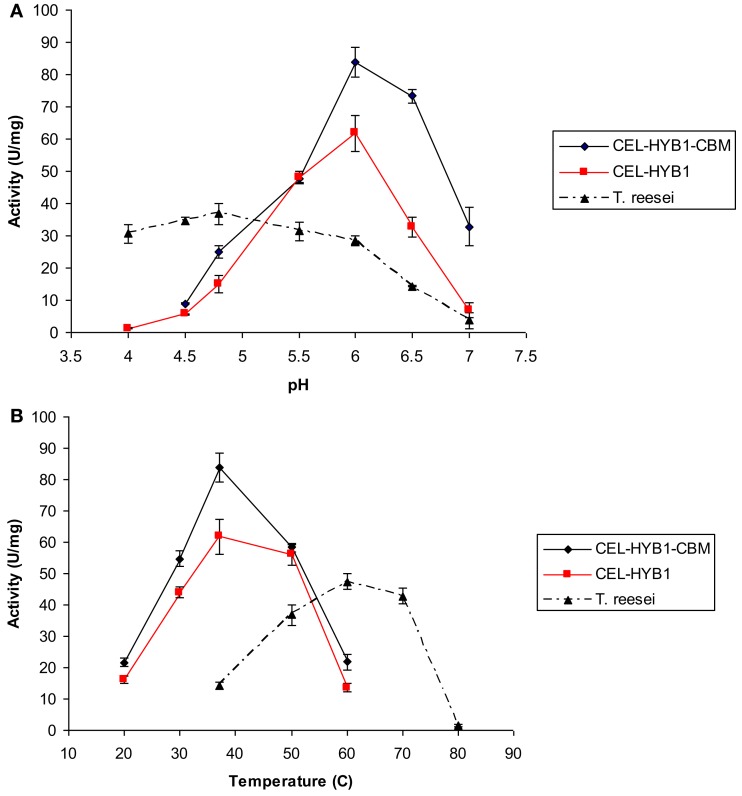
**CEL-HYB1 and CEL-HYB1-CBM enzyme activity at varying temperature and pH**. Effects of pH **(A)** and temperature **(B)** on the endo-β-1,4-glucanase activity of CEL-HYB1, CEL-HYB1-CBM, and *T. reesei* cellulase enzymes. Measurements shown in **(A)** were performed at 37°C for CEL-HYB1 and CEL-HYB1-CBM, and 50°C for the *T. reesei* cellulase cocktail. **(B)** Measurements were obtained using optimal pH for each enzyme as shown in **(A)**. Measurements were performed in triplicate. The activity is expressed as μmol of reducing sugar min^−1^ (U) mg^−1^. The error bars represent standard deviation.

The optimal pH and temperature conditions for CEL-HYB1 and CEL-HYB1-CBM endo-β-1,4-glucanase activity were determined using low viscosity CMC as the substrate and measuring the concentration of reducing sugar produced by the DNS assay (Figures 3A,B). The optimal hydrolysis conditions for the *Trichoderma reesei* cellulase enzyme cocktail (ATCC26921, Sigma), composed of multiple cellulase enzymes, was also investigated as a comparison (Figures 3A,B). The commercial *T. reesei* cellulase enzyme mixture contains at least two cellobiohydrolases (CBHI and II), four endo-β-1,4-glucanases (EGI, II, III, and V), and two β-1,4 glucosidases (BGLI and II; Seiboth et al., 1997). The cellulase enzyme cocktail derived from *T. reesei* was used as a comparator for the activity of the synthetic cellulase enzymes as cellulases from *T. reesei* are commonly used in bioethanol production facilities (Margeot et al., 2009).

The hydrolytic activity of CEL-HYB1 and CEL-HYB1-CBM on CMC was optimal at pH 6.0 (Figure 3B), with almost no endo-β-1,4-glucanase activity measured at pH 4.0. The hydrolytic activity of the cellulase enzyme cocktail from *T. reesei* was most active at pH 4.8 and in contrast to the CEL-HYB1 and CEL-HYB1-CBM enzymes, the activity was found to be largely unaffected by pH, especially between pH 4.0 and 5.5.

Once the optimal pH for CEL-HYB1, CEL-HYB1-CBM, and the *T. ressei* cellulase enzyme cocktail was determined, the effect of temperature on the activity of the enzymes was investigated at optimal pH. The highest CEL-HYB1 and CEL-HYB1-CBM endo-β-1,4-glucanase activity was measured at 37°C whereas the *T. reesei* cellulase cocktail demonstrated maximal activity at 60°C, although stability was evident across a broad temperature range between 50 and 70°C.

**Figure 4:**
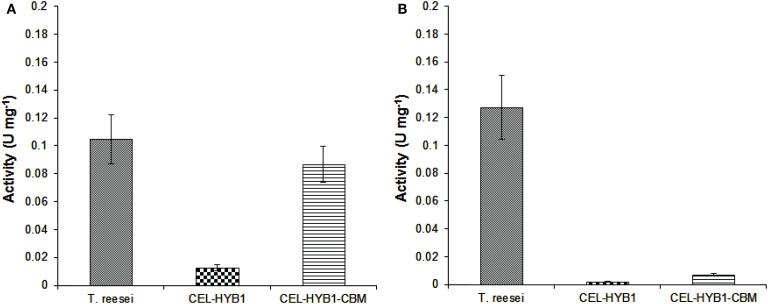
**Endo-and exo-glucanase activity of CEL-HYB1 with and without the CBM**. The endo-β-1,4-glucanase **(A)** and exo-β-1,4-glucanase activity **(B)** of CEL-HYB1, CEL-HYB1-CBM, and the *T. reesei* cellulase enzyme cocktail (U mg^−1^ ± SD) were compared. Measurements shown in **(A)** were determined using acid-pre-treated Sorghum material as the substrate, whereas measurements shown in **(B)** were determined using the MUC assay. The measurements were repeated three times. The activity is expressed as μmol of reducing sugar min^−1^ (U) mg^−1^ for the Sorghum assay, whereas the result of MUC activity assay is expressed as μmol of MU min^−1^ (U) mg^−1^. The endo-β-1,4-glucanase assay was performed at 37°C and pH 6.0 for CEL-HYB1 and CEL-HYB1-CBM, whereas for *T. reesei*, cellulose enzyme measurement was carried out at 60°C and pH 4.8. The MUC assay was performed at 37°C and pH 6.0 for all enzymes. Values with the same letters are not different significantly at *P* < 0.05 (Fisher’s LSD test).

The capacity of CEL-HYB1, CEL-HYB1-CBM, and *T. reesei* cellulase enzymes to hydrolyze native crystalline cellulose was investigated using pre-treated Sorghum stem material as the substrate and the optimal conditions for each respective enzyme. The *T. reesei* cellulase enzyme cocktail exhibited the highest hydrolytic capacity (0.105 U mg^−1^ ± 0.018) followed very closely by CEL-HYB1-CBM (0.087 U mg^−1^ ± 0.013). The CEL-HYB1, however, showed very low hydrolytic activity (0.013 U mg^−1^ ± 0.002), indicating that without the CBM the enzyme was not very efficient in hydrolyzing crystalline cellulose (Figure 4A).

In addition to the CMC and pre-treated sorghum assay, an MUC assay was also performed to investigate the capacity of the CEL-HYB1 and CEL-HYB1-CBM enzymes to cleave the β-1,4 bonds located at the end of the polysaccharide chain (Schwarz et al., 1987). The assays were performed at 37°C and pH 6.0. The exo-glucanase activity was then directly compared to the exo-glucanase activity of the *T. reesei* cellulase enzyme cocktail measured under the same conditions. The CEL-HYB1 and CEL-HYB1-CBM MUC activity was calculated to be 0.0019 ± 0.00015 and 0.0072 ± 0.001 U mg^−1^, respectively, whereas the MUC activity of the *T. reesei* cellulase enzyme cocktail was determined to be 0.128 ± 0.023 U mg^−1^ (Figure 4B). The CEL-HYB1-CBM demonstrated almost a fourfold higher exo-glucanase activity as compared with the CEL-HYB1, although activity was inconsequential as compared with the *T. reesei* enzyme cocktail.

## Discussion

### Plant CBM improves glycosyl hydrolase enzyme activity

The tomato CBM at the C-terminus of the synthetic microbial enzyme, CEL-HYB1, increased the endo-β-1,4-glucanase activity of the enzyme across a range of temperatures and pH (Figure 3). The presence of the CBM, however, did not shift the hydrolytic characteristics of the enzyme, indicating that the increased CEL-HYB1-CBM activity may be attributed to the increased enzyme affinity toward the substrate, CMC.

In addition to the CMC assay, the activity of the synthetic enzymes was tested using pre-treated Sorghum plant material. The CMC assay does not measure the absolute endo-β-1,4-glucanase activity as the substrate used is non-crystalline cellulose and hence, the assay was used as an indicator of cellulase activity whilst optimizing conditions for the assay. Alternatively, the activity of cellulase enzymes against crystalline cellulose may be tested using the Avicel assay which utilizes microcrystalline cellulose (Avicel) as the substrate (Taylor et al., 2008). For example, the activity of the *Ruminococcus albus* Cel5 on CMC did not differ significantly when fused with a *Clostridium stercorarium* CBM yet the activity of the same Cel5:CBM fusion protein on Avicel was enhanced compared to the native Cel5 (Bae et al., 2003). Interestingly, the activity of the *Clostridium thermocellum* endoglucanase (EGE_CD_) on CMC did not differ significantly when fused with a *Pseudomonas fluorescens* cellulose binding domain (CELE_CBD_) or xylanase binding domain (XYLA_CBD_) yet the activity of the EGE_CD_:CELE_CBD_ and EGE_CD_:XYLA_CBD_ fusion proteins on cotton was significantly enhanced compared to EGE_CD_. In this case the activity of the fusion proteins was significantly lower on Avicel relative to EGE_CD_ (Bolam et al., 1998). Although the Avicel assay may be a better method to estimate the cellulase activity as compared with the CMC assay, it is still limited as the interaction of cellulases with other plant cell wall components such as lignin, may affect the overall activity of the enzymes and this would not be represented. Thus, the activity of the synthetic enzymes against crystalline sorghum cellulose from pre-treated stem biomass was undertaken to provide an estimate of enzyme activity on native plant cellulose.

In comparison with the CMC assay results, when the acid-pre-treated Sorghum plant material was used as the substrate, all of the tested enzymes demonstrated lower endo-β-1,4-glucanase activity. The CEL-HYB1 had very low activity compared to the *T. reesei* cellulase cocktail. The presence of the CBM on the CEL-HYB1 improved the activity dramatically such that the endo-β-1,4-glucanase activity of the fusion protein was similar to that of the *T. reesei* cellulase cocktail. The *T. reesei* cellulase enzyme cocktail was a mixture of several cellulase enzymes which included endo-β-1,4-glucanase, exo-glucanase, and β-glucosidase and it has been demonstrated that when present together different cellulase enzymes may have some level of synergism, resulting in improved hydrolytic activity (Irwin et al., 1993; Hilden and Johansson, 2004). Additionally, all endo-β-1,4-glucanase and exo-β-1,4-glucanase enzymes derived from *T. reesei* are reported to have CBMs and hence the enzymes would be expected to catalyze increased hydrolytic activity (Takashima et al., 1998).

### Enzyme activity on plant biomass differs from activity on commercial cellulose

CEL-HYB1-CBM is active against the Sorghum crystalline cellulose as is; however, mixing of the enzyme with exo-glucanase and β-glucosidase is likely to improve the activity further. The stark differences between the results of the CMC assay compared with the use of pre-treated sorghum as a substrate simply highlight the inadequacy of the CMC assay as a tool to predict cellulase activity against crystalline cellulose.

The increase in CEL-HYB1-CBM activity can be attributed to the presence of the CBM which potentiates the binding capability of the enzyme to crystalline cellulose. This assertion is supported by data presented previously demonstrating that the CBM of SlCel9C1 is able to bind to crystalline cellulose (Urbanowicz et al., 2007). Previous reports have shown that cellulase activity against non-soluble cellulose could be increased significantly by adding a microbial CBM to the enzyme (Bae et al., 2003; Moses et al., 2005; Mahadevan et al., 2008).

### Engineering more active enzymes

The results of the CMC and sorghum hydrolysis indicate that a CBM originating from an endogenous plant cellulase can have a positive impact on the hydrolytic capacity of the enzyme. An earlier attempt to increase the enzyme hydrolytic capability by attaching the CBM of SlCel9C1 to the C-terminus of TfCel6A, an endo-β-1,4-glucanase from *Thermobifida fusca*, failed as the fusion protein demonstrated lower activity as compared with the native enzyme (Urbanowicz et al., 2007). This was hypothesized to be caused by the configuration of the fusion protein, whereby the CBM module separated the CD of the enzyme from the substrate (Urbanowicz et al., 2007). The results presented herein demonstrate that the presence of the CBM at the C-terminus of CEL-HYB1 did not hinder the binding of the substrate to the active site of the cellulase even though no linker region between the two domains was present.

As native plant cellulase enzymes are involved in re-modeling the cell wall during growth it is likely that there is great variation in the substrate affinity within plant CBMs. As mixtures of cellulase enzymes can have synergistic effects (Irwin et al., 1993), the impact of fusing various plant CBMs to a range of highly active endo- and exo-glucanases and assessing the activity of different mixtures of these fusion proteins would be of interest.

As demonstrated here, the activity of cellulases may be altered by recombining regions of natural cellulase homologs. This approach was also used to demonstrate the importance of a CBM to the thermal stability of a family 9 cellulase (Mingardon et al., 2011). An alternative option for increasing the activity of cellulase enzymes may involve mutating the linker region between the CD and the CBM so as to alter the flexibility; this may influence conformational changes and thus activity (Batista et al., 2011). There is also much to learn about the impact of glycosylation on the function of CBMs (Beckham et al., 2012). CBMs and their linkers may have *O*-glycans attached, whereas the CD may have *N*-glycans attached; these glycans may interact directly with the cellulose and impact binding affinity (Beckham et al., 2012). Thus, modifying cellulose enzyme and CBM glycosylation may be another avenue toward improving enzyme activity.

## Conflict of Interest Statement

The authors declare that the research was conducted in the absence of any commercial or financial relationships that could be construed as a potential conflict of interest.
